# Deleterious Rare Mutations of *GLI1* Dysregulate Sonic Hedgehog Signaling in Human Congenital Heart Disease

**DOI:** 10.3389/fcvm.2022.798033

**Published:** 2022-04-04

**Authors:** Rui Peng, Binbin Li, Shuxia Chen, Zhiwen Shi, Liwei Yu, Yunqian Gao, Xueyan Yang, Lei Lu, Hongyan Wang

**Affiliations:** ^1^NHC Key Laboratory of Reproduction Regulation, State Key Laboratory of Genetic Engineering, Obstetrics and Gynecology Hospital, Shanghai Institute of Planned Parenthood Research, Institute of Reproduction and Development, Children's Hospital, Fudan University, Shanghai, China; ^2^Shanghai Key Laboratory of Metabolic Remodeling and Health, School of Life Sciences, Institute of Metabolism and Integrative Biology, Fudan University, Shanghai, China; ^3^Department of Molecular, Cellular, and Developmental Biology, University of Colorado Boulder, Boulder, CO, United States; ^4^Center for Craniofacial Molecular Biology, University of Southern California, Los Angeles, CA, United States; ^5^SUNY Downstate Medical Center, Children's Hospital at Downstate, Brooklyn, NY, United States; ^6^Department of Laboratory Medicine, Shanghai Children's Medical Center, Shanghai Jiao Tong University School of Medicine, Shanghai, China

**Keywords:** congenital heart disease (CHD), *GLI1*, genetic variant, rare mutation, Sonic hedgehog (SHH) signaling pathway

## Abstract

The Glioma-associated oncogene (Gli) family members of zinc finger DNA-binding proteins are core effectors of Sonic hedgehog (SHH) signaling pathway. Studies in model organisms have identified that the *Gli* genes play critical roles during organ development, including the heart, brain, kidneys, *etc*. Deleterious mutations in *GLI* genes have previously been revealed in several human developmental disorders, but few in congenital heart disease (CHD). In this study, the mutations in *GLI1-3* genes were captured by next generation sequencing in human cohorts composed of 412 individuals with CHD and 213 ethnically matched normal controls. A total of 20 patient-specific nonsynonymous rare mutations in coding regions of human *GLI1-3* genes were identified. Functional analyses showed that *GLI1* c.820G> T (p.G274C) is a gain-of-function mutation, while *GLI1* c.878G>A (p.R293H) and c.1442T>A (p.L481X) are loss-of-function mutations. Our findings suggested that deleterious rare mutations in *GLI1* gene broke the balance of the SHH signaling pathway regulation and may constitute a great contribution to human CHD, which shed new light on understanding genetic mechanism of embryo cardiogenesis regulated by SHH signaling.

## Introduction

Congenital heart disease (CHD) is the most common developmental anomaly and the leading non-infectious cause of mortality in newborns (1). In China, the incidence of CHD has been increased from 0.201‰ in 1980–1984 to 4.905‰ in 2015–2019 (2). Ventricular septal defect (VSD) and atrial septal defect (ASD) are the most common congenital heart defects subtypes among the offspring. The etiology of CHD is complicated, and genetic factors play important roles in CHD occurrence (3–5). However, identification of these factors has been historically slow due to technical limitations and short understanding of signaling pathways regulating embryonic cardiovascular development (5, 6).

In mammals, the Hedgehog (HH) family genes of Sonic hedgehog (SHH), Indian hedgehog (IHH) and Desert hedgehog (DHH) encode evolutionarily conserved ligand proteins initiating pathways crucial for embryogenesis (7). The SHH signaling pathway is transduced by the seven-transmembrane G-protein-coupled receptor (GPCR)-like protein Smoothened (Smo), leading to the activation of Glioma-associated oncogene (Gli) family of transcription factors and downstream target genes transcription (8–10). In vertebrates SHH signaling pathway, *Gli* gene family contains three members of *Gli1, Gli2*, and *Gli3* (11). Gli1 protein acts as a transcriptional activator and provides a positive feedback loop of signaling, whereas Gli2 and Gli3 serve as both transcriptional activators and repressors, depending on specific post-translational modifications and proteolysis processes (12, 13).

SHH signaling pathway has recently been implicated in the specification of early embryonic cardiac progenitor fate. SHH signaling specifies atrial septum from non-septum atrial progenitors (14). Dysregulated SHH signaling pathway involves in numerous human diseases, including birth defects and cancers (15). Various cardiac malformations are observed in *Shh*^−/−^ mouse embryos (16). The critical roles of *GLI1-3* in embryonic development have been well established (17, 18). *GLI1* participates in differentiation and development of several organs in humans through SHH signaling pathway (19, 20). According to a recent study, *GLI1* nonsynonymous variants were identified among 25 heterotaxy syndrome (HS) patients with CHD (21). Moreover, *Gli2*^−/−^ and *Gli3*^+/−^ double mutant mice show a full complement of VACTERL syndrome including cardiac defects (22). These results raise the possibilities that functional rare mutations in *GLI1-3* may also affect human embryo cardiogenesis.

To understand the association between *GL1-3* variants and the risk of CHD in humans, we screened 5′-untranslated region (UTR), 3′-UTR and coding regions of *GLI1-3* genes in a Chinese cohort with 412 cases and 213 matched controls by next generation sequencing. As a result, a total of 20 patient-specific nonsynonymous rare mutations in coding regions of human *GLI1-3* genes were identified. Our *in vitro* and *in vivo* functional analyses showed that *GLI1* c.820G>T (p.G274C) is a gain-of-function mutation, *GLI1* c.878G>A (p.R293H) and c.1442T>A (p.L481X) are loss-of-function mutations. Thus, our data implicate the association between dysregulated SHH signaling pathway and CHD occurrence, and broaden the current knowledge of human embryonic cardiogenesis.

## Materials and Methods

### Human Samples

Sample collection was performed as described previously (23–25). Blood samples from 412 CHD patients (mean age 2.9 ± 2.7 years, 55.6% males) were collected from the Cardiovascular Disease Institute of Jinan Military Command (Jinan, China). The patients were diagnosed by echocardiography, and some were further confirmed surgically. Patients with clinical features of developmental anomalies, positive family history of CHD in a first-degree relative, maternal diabetes mellitus, maternal exposure to known teratogens or any therapeutic drugs during gestation were excluded. The 213 controls (mean age 7.1 ± 3.7 years, 49.8% males) were ethnically and gender-matched, unrelated healthy volunteers recruited from the same geographical area. Sample collection and protocols used in this study were reviewed and approved by the Ethics Committee of the School of Life Sciences, Fudan University and local ethics committees before the start of the present study. All procedures were in accordance with the Declaration of Helsinki. Informed consents were signed by the parents or guardians of the children. Detailed information of the samples was shown in Table 1.

**Table 1:** Information of CHD cases and controls.

**Variable**	**Case (%)**	**Control (%)**
Sequencing group	412	213
Region	Shandong	Shandong
Age: years (mean ± S.D.)	2.9 ± 2.7	7.1 ± 3.7
Male [no. (%)]	229 (55.6)	106 (49.8)
Female [no. (%)]	183 (44.4)	107 (50.2)
**CHD classification**		
Septation defects [no. (%)]	136 (33.0)	
TOF^a^ [no. (%)]	72 (17.5)	
AVSD^b^ [no. (%)]	64 (15.5)	
DORV^c^ [no. (%)]	39 (9.5)	
PDA^d^ [no. (%)]	29 (7.0)	
APVR^e^ [no. (%)]	11 (2.7)	
TAPVC^f^ [no. (%)]	9 (2.2)	
Others^g^ [no. (%)]	52 (12.6)	

a *TOF, Tetralogy of Fallot*.

b *AVSD, Atrioventricular septal defect*.

c *DORV, Double-outlet right ventricle*.

d *PDA, Patent ductus arteriosus*.

e *APVR, Anomalous pulmonary venous return*.

f *TAPVC, Total anomalous pulmonary venous connection*.

g *“Others” includes CHDs with other defects such as heterotaxy*.

### DNA Sequencing and Data Analysis

Peripheral blood samples were collected. Genomic DNA was extracted and target-sequenced was conducted as described previously (23–25). The genomic structures of human *GLI1-3* genes were determined using NCBI Genebank (mRNA references are NM_001166045, NM_005270, NM_000168, respectively). The 5′ -UTR, 3′ -UTR and coding regions in *GLI1-3* were screened. Identified variants were filtered using the dbSNP database (http://www.ncbi.nlm.nih.gov/projects/SNP), the 1000 genomes projects (http://www.1000genomes.org/), the Genome Aggregation Database (gnomAD, http://gnomad-sg.org/), and the HuaBiao Database (https://www.biosino.org/wepd) (26). All the patient-specific nonsynonymous mutations were confirmed by Sanger sequencing, the PCR primers were listed in Supplementary Table S1.

### Plasmids

Human *GLI1*-*3* ORF without stop codon were amplified by PCR (Supplementary Table S2) using cDNA reverse transcripted from human total RNA. *GLI1* and *GLI3* were subcloned into SgfI/MluI restriction enzyme sites. *GLI2* was subcloned into SgfI/NotI restriction enzyme sites of pCMV6-AC-HA (Origene, #PS100004). All plasmid were verified by Sanger sequencing. We then performed the site-directed mutagenesis using QuikChange Lightning Site-Directed Mutagenesis Kit (Agilent, #210518) according to manufacturer's instruction. For *GLI1* c.1442T>A (p.L481X) stop-gain mutation, sequence after premature stop codon (including stop codon) was removed for C-terminal tag fusion. The pGMGLI-Lu firefly luciferase SHH signaling pathway reporter plasmid was obtained from Genomeditech (#GM-021024). The constitutive Renilla luciferase reporter pRL-TK was from Promega (#E6921).

### Cell Culture and Transfection

HEK 293T cells (ATCC, #CRL-3216) and HeLa cells (ATCC, #CCL-2) were cultured in high-glucose Dulbecco's Modified Eagle Medium (DMEM, Thermo Fisher Scientific, #11995065) supplemented with 10% FBS (Thermo Fisher Scientific, #A3840001) at 37°C with 5% CO_2_. The cells were seeded and maintained overnight to reach ~80% confluency at the time of transfection by Lipofectamine 2000 Transfection Reagent (Thermo Fisher Scientific, #11668019) and Lipofectamine 3000 Transfection Reagent (Thermo Fisher Scientific, #L3000015) separately according to manufacturer's protocols.

### Dual-Luciferase Reporter Assay

Dual-luciferase reporter assay was performed as described previously (24, 27). Briefly, in a 24-well plate well, 300 ng of empty vector or *GLI1* wild-type/mutant constructs, 200 ng of pGMGLI-Lu firefly luciferase reporter plasmid, 10 ng of Renilla luciferase plasmid serving as an internal control were transfected. Reporter assay was performed with the Dual-Luciferase Reporter Assay System (Promega, #E1910) on GloMax Navigator Microplate Luminometer (Promega, #GM2010) 24 h post transfection. At least three independent biological repeats were performed, and data were presented as the mean ± S.D. *P-*values were calculated by Student's *t*-test and considered significant when <0.05.

### Western Blotting

C-terminal HA-tagged wild-type or mutant *GLI1* expressing plasmids were transfected into HEK293T cells. Forty-eight hours later, cells were lysed with Cell lysis buffer for Western and IP (Beyotime, #P0013) containing a cocktail of protease inhibitors (ThermoFisher Scientific, #1862209) and heated for 10 min at 100°C. Cell lysates were loaded and separated on a 10% SDS-polyacrylamide gel electrophoresis and transferred onto a PVDF membrane (Merck Millipore, #ISEQ00010). After blocking for 1 h with 5% non-fat milk, the membrane was incubated with mouse HA-tag antibody (Proteintech, #66006-2-Ig) and mouse anti-GAPDH antibody (Proteintech, #66004-1-Ig) at 4°C overnight. Horseradish peroxidase-conjugated anti-mouse IgG was served as secondary antibody (Cell signaling Technology, #7076) for 2 h at room temperature and visualized through the ECL Detection System (Tanon, Shanghai, China) (25). Three independent experiments were performed and representative results were shown.

### Electrophoretic Gel Mobility Shift Assay (EMSA)

The probes (F: 5′-AGCTACCTGGGTGGTCTCT-3′, R: 5′-TCGAAGAGACCACCCAGGT-3′) were designed according to the consensus GLI-binding site (5′-TGGGTGGTC-3′) from *PTCH1* promoter region (Supplementary Table S3) (28). The DNA-binding affinity of the different GLI proteins was determined by EMSA according to previous description (29). Briefly, nuclear extracts were prepared from HEK 293T cells transfected with empty vector or *GLI1* wild-type/mutation constructs using the NE-PER Nuclear and Cytoplasmic Extraction kit (ThermoFisher Scientific, #78835) and stored at −80°C before use. Protein concentrations were determined by Pierce BCA Protein Assay Kit (ThermoFisher Scientific, #23227). Nuclear proteins were mixed and incubated with indicated probes and subsequently processed using LightShift Chemiluminescent EMSA Kit (ThermoFisher Scientific, #20148) following manufacturer's instruction.

### Immunofluorescence

HeLa cells were plated into 35 mm glass bottom dishes and cultured overnight to reach ~80% confluency. Then cells were transiently transfected with HA-tagged *GLI1* wild-type, p.G274C, p.R293H, or p.L481X constructs. 24 h later, cells were fixed in 4% PFA for 15 min at room temperature, then immunofluorescence was performed using HA antibody (MilliporeSigma, #H6908) as described previously (25), and images were captured on a Zeiss LSM700 confocal microscope under 40x objective lens. Experiment was repeated at least in triplicates and representative result was presented.

### Zebrafish Embryo Microinjection

Wild-type AB and Cmlc2-mCherry strain zebrafish (Danio rerio) were maintained under standard conditions (25, 30). All plasmids of the empty vector, *GLI1* wild-type, p.G274C, p.R293H, and p.L481X were extracted by Endo-free Mini Plasmid Kit II (Tiangen Biotech, #DP118), and diluted in nuclease-free water. In this study, each plasmid was injected into >200 zebrafish embryos at 1–2 cell stage. From each plasmid 2–3 nl was injected at a concentration of 40 ng/μl. 72 h post injection, photographs were taken using Leica MZ95 stereo microscope (30) or Olmpus IX83 fluorescence inverted microscope and the percentages of pericardial abnormal embryos were calculated (25). Phenotype distribution differences compared with wild-type group were calculated using χ2 analysis (25).

## Results

### Identification of Variants in *GLI1*-*3* Genes of CHD Patients

A Chinese CHD cohort with ethnically and gender-matched healthy controls was used in this study. The 5′-UTR, 3′-UTR and coding regions of *GLI1-3* genes were sequenced in all patients and controls. CHD is one of severe disorders that impacts mortality and reproductive fitness. A very large negative selection eliminates highly deleterious common mutations from the human population. Thus the existing common variants like single nucleotide polymorphism (SNP) may make less contributions to this disease. Therefore, it is most likely that novel nonsynonymous rare mutations make significant contributions to population prevalence of this defect, especially for sporadic cases. Based on this hypothesis, we sorted out the variants with minor allele frequencies (MAF) < 1% as rare variants.

**Figure 1:**
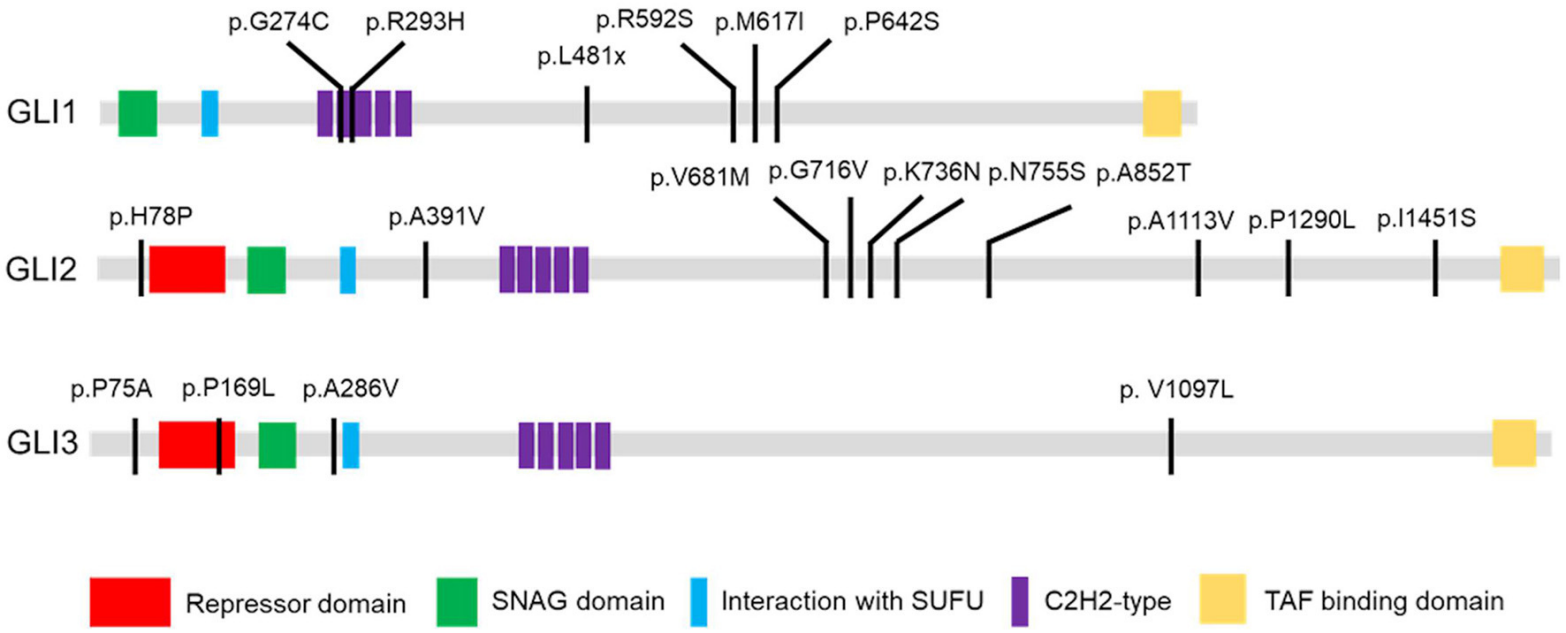
GLI1-3 mutations were identified in CHD patients. Schematic diagram of case-specific mutations in GLI1, GLI2 and GLI3 protein.

A total of 94 variants in the *GLI1-3* genes were identified in CHD patients (these data were not shown). 20 nonsynonymous patient-specific rare mutations were identified (Table 2, Figure 1) and further confirmed by Sanger sequencing (Supplementary Figure S1). The effect of rare missense variants on the protein function was predicted *in silico* using SIFT, PolyPhen-2 and CADD (Table 2) (31–34). We also evaluated the evolutionary conservativeness of these mutations with Genomic Evolutionary Rate Profiling (GERP, the higher of the GERP score means that the site is more conserved) (Table 2) (35), as well as alignment assay across several species (Supplementary Figure S2). For *GLI1* gene, we identified one nonsense stop-gain rare mutation and five missense rare mutations. Of which, the stop-gain mutation of c.1442T>A (p.L481X) leads to loss of several crucial domains including the transcriptional activation domain. Mutations of c.820G>T (p.G274C), c.878G>A (p.R293H), c.1776A>T (p.R592S) and c.1924C>T (p.R642S) are predicted as harmful mutations by both SIFT and PolyPhen-2. In addition, mutations of p.G274C and p.R293H locate in the zinc finger domain, which probably interfere with the protein-DNA affinity.

**Table 2:** Bioinformatics analysis of patient-specific nonsynonymous rare mutations identified in *GLI1-3*.

**Case**	**Age**	**Sex**	**Phenotype**	**Gene**	**Position**	NA variant^*^	**AA variant**	**Type of variant**	**GERP score**	MAF in gnomAD^**^	**MAF in HuaBiao**	**SIFT^a^**	**PP2^b^**	**CADD^c^**
#1	0.6	F	VSD,ASD	*GLI1*	chr12:57860080	c.820G>T	p.G274C	Missense	4.93	0.00003184	NA	D	PrD	P
#2	8	F	TOF	*GLI1*	chr12:57860138	c.878G>A	p.R293H	Missense	4.93	NA	0.0001	D	PrD	P
#3	6	F	ASD, PDA, PH	*GLI1*	chr12:57863347	c.1442T>A	p.L481X	Stop-gain	4.66	NA	NA	NA	NA	P
#4	9.25	F	ASD, PS	*GLI1*	chr12:57864299	c.1776A>T	p.R592S	Missense	−0.05	NA	NA	D	PrD	P
#5	7.25	F	VSD, ASD, PDA	*GLI1*	chr12:57864374	c.1851G>A	p.M617I	Missense	−0.43	0.00001195	NA	T	B	B
#6	1.58	F	ASD, PDA	*GLI1*	chr12:57864447	c.1924C>T	p.P642S	Missense	4.44	0.000004010	NA	D	PrD	P
#7	1.17	M	AVCD, TAPVC, DORV	*GLI2*	chr2:121685021	c.233A>C	p.H78P	Missense	1.56	NA	NA	T	PrD	P
#8	1.17	M	VSD, PS, DORV	*GLI2*	chr2:121729629	c.1172C>T	p.A391V	Missense	−0.31	0.00001066	NA	T	B	B
#9	3.33	F	AVCD, PH	*GLI2*	chr2:121743938	c.2041G>A	p.V681M	Missense	−2.72	0.00007115	0.0001	D	B	P
#10	6.75	F	VSD, ASD, PDA	*GLI2*	chr2:121744044	c.2147G>T	p.G716V	Missense	0.81	0.000003988	NA	T	PrD	P
#11	1.75	F	ASD	*GLI2*	chr2:121744105	c.2208G>C	p.K736N	Missense	1.97	0.00005313	0.0004	D	PrD	P
#12	5	F	PDA	*GLI2*	chr2:121744161	c.2264A>G	p.N755S	Missense	−3.54	NA	NA	T	B	B
#13	3	M	VSD	*GLI2*	chr2:121746044	c.2554G>A	p.A852T	Missense	2.91	0.00003428	0.0004	T	PrD	P
#14	0.25	F	TAPVC	*GLI2*	chr2:121746828	c.3338C>T	p.A1113V	Missense	1.94	NA	NA	T	B	B
#15	7	M	DORV, PS	*GLI2*	chr2:121747359	c.3869C>T	p.P1290L	Missense	2.76	NA	NA	T	B	B
#16	11	M	VSD	*GLI2*	chr2:121747842	c.4352T>G	p.I1451S	Missense	4.22	NA	NA	T	PrD	P
#17	1.92	M	CoA	*GLI3*	chr7:42187969	c.223C>G	p.P75A	Missense	5.07	0.00004245	0.0001	T	PrD	P
#18	0.25	F	AVCD	*GLI3*	chr7:42088263	c.506C>T	p.P169L	Missense	4.91	0.00001593	0.0001	D	B	P
#19	0.92	F	AVCD	*GLI3*	chr7:42079808	c.857C>T	p.A286V	Missense	4.13	0.000007072	NA	T	B	P
#20	3	M	VSD	*GLI3*	chr7:42005382	c.3289G>T	p.V1097L	Missense	4.58	NA	NA	T	PrD	P

*VSD, Ventricular septal defect; ASD, Atrial septal defect; PH, Pulmonary hypertension; PS, Pulmonary stenosis; AVCD, Atrioventricular canal defect; CoA, Coarctation of aorta*.

* *All the variants are heterozygous*.

** *All of the variants we identified are heterozygous in gnomAD*.

a *SIFT predictions: D represents damaging, T represents tolerated*.

b *PolyPhen-2 (PP2) predictions: B represents benign, PrD represents probably damaging*.

c *CADD predictions: P represents pathogenic, B represents benign*.

### Three *GLI1* Mutations Had Significantly Different Activities Compared to Wild-Type *GLI1*

**Figure 2:**
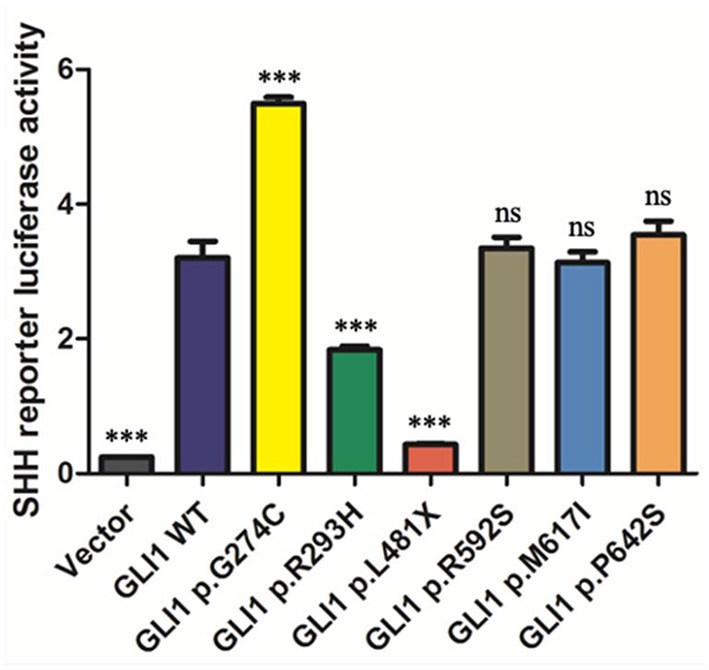
The statistical analyses of luciferase relative activity for HEK293T cells co-transfected with expressing plasmids as indicated and Sonic hedgehog (SHH) signaling pathway reporter plasmids. Activation of SHH signaling pathway pGMGLI-Lu firefly luciferase reporter by human GLI1 wild-type or mutants 24 h post-transfection in HEK 293T cells. Constitutively expressed Renilla Reniformis Luciferase served as an internal control (n ≥ 3, *P < 0.05, **P < 0.01, ***P < 0.001, ns, not significant, compared with wild-type group).

As GLI1 protein only serves as transcriptional activator whereas GLI2 and GLI3 can be processed into both transcriptional activator and repressor under specific condition, we first put our attentions on studying GLI1 mutations. To explore the effect of these nonsynonymous rare variants of *GLI1* on regulating SHH signaling pathway, we conducted dual-luciferase reporter assay using pGMGLI Lu reporter containing GLI binding sites upstream of firefly luciferase, and Renilla luciferase plasmid as internal control. As expected, wild-type *GLI1* dramatically activates the signaling compared with the empty vector group, whereas the p.G274C mutation shows increased activation, in contrast, p.R293H and p.L481X result in significantly decreased activation of the signaling in HEK293T cells. The result indicates that p.G274C is a gain-of-function mutation, whereas p.R293H and p.L481X are loss-of-function mutations. As predicted, the nonsense mutation p.L481X generating a truncated protein (Supplementary Figure S3) almost completely loses its ability in SHH signaling pathway activation (Figure 2).

### p.G274C Mutation *GLI1* Protein Enhance the Protein Binding Affinity With DNA

**Figure 3:**
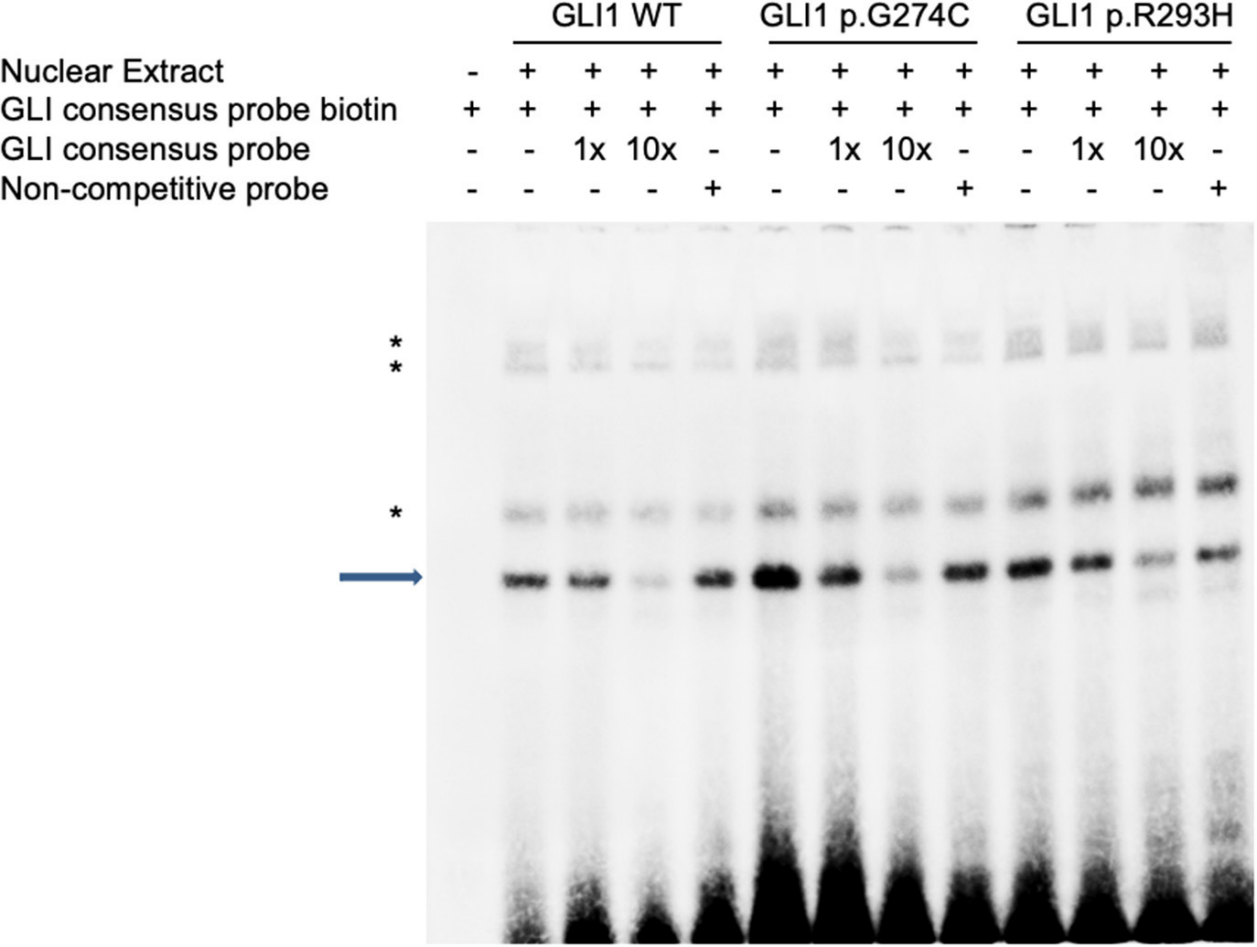
GLI1 mutations affect protein binding affinity with DNA in Electrophoretic Mobility Shift Assay (EMSA). HEK293T cells were transfected with wild-type or mutant GLI1 expression constructs. Nuclear extract was prepared and incubated with specific DNA oligos as indicated. Arrow highlights specific GLI1-DNA binding signal. Asterisks represent non-specific signals.

As there is no doubt that the nonsense mutation of p.L481X disrupts protein function due to the loss of large portion of several critical domains (Figure 1), we focused on the other two harmful missense mutations of p.G274C and p.R293H as indicated above. Immunoblot analysis revealed no significant protein stability change made by two mutations (Supplementary Figure S3). Both mutations are mapped into the zinc finger domain of GLI1 protein (Figure 1), raising the possibility that they probably affect the protein binding ability with DNA. To this end, we performed electrophoretic mobility shift assay (EMSA) using nuclear extract from *GLI1* wild-type/mutation constructs transfected cells and synthesized probes containing GLI protein bind motif from *PTCH1* promoter. The result demonstrates that, the mutation of p.G274C results in significantly higher binding ability with GLI-Probe than wild-type (Figure 3), which probably accounts for its enhanced SHH signaling activation. Slightly higher binding ablility with GLI-Probe was also found in p.R293H (Figure 3).

### The Mutation p.L481X Affects GLI1 Protein Subcellular Localization

**Figure 4:**
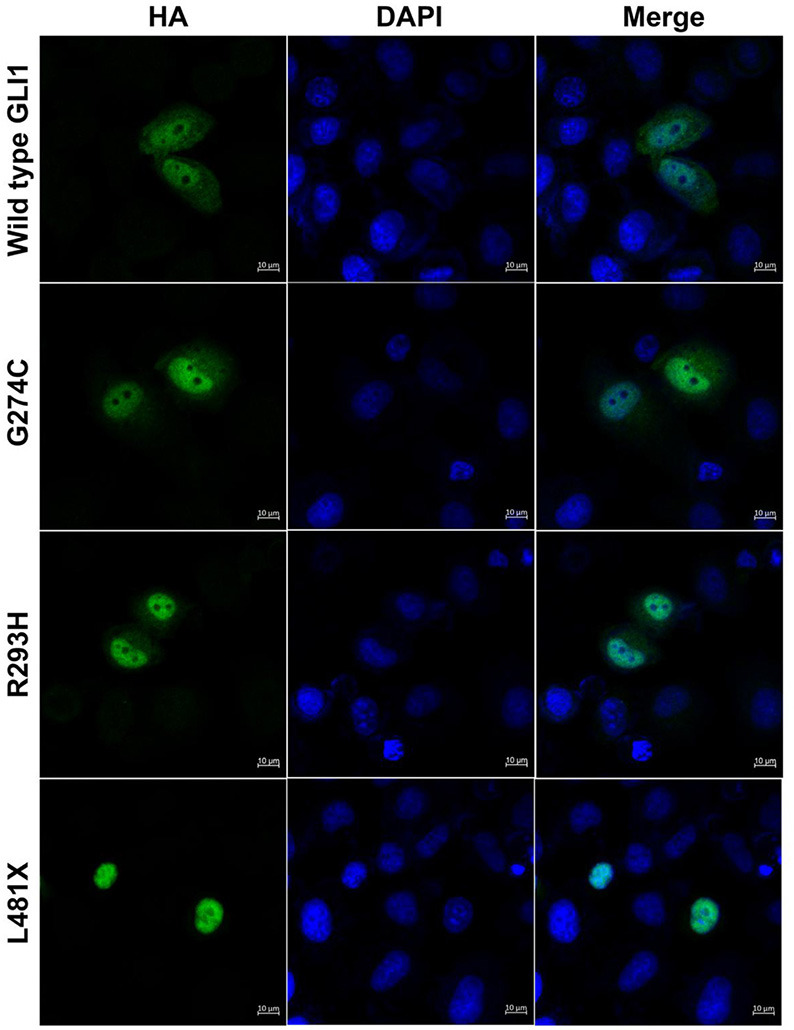
GLI1 p.L481X affect protein subcellular localization. HeLa cells were transfected with HA-tagged wild-type or mutant *GLI1* expression constructs. Immunofluorescence was performed using anti-HA antibody (Green). Nuclei were counterstained with Hoechst (Blue). Scale bars are 10 μm.

GLI1 protein has two putative nuclear localization signals (NLS), including a monopartite signal NLS1 (aa 79-84) and a bipartite signal NLS2 (aa 383-401) (36). In addition, GLI1 possesses a leucine-rich nuclear export signal (NES) (aa 496-504), which is fully conserved in other vertebrate species (36, 37). In order to evaluate the functional consequences of the mutations, HeLa cells were transfected using Lipofectamine 3000 with various *GLI1* plasmid constructs. No significant differences in level of expression were detected between these GLI1 variants. Both proteins were readily detectable in the nucleus of transfected cells (Figure 4). However, compared to wild-type GLI1 protein, p.L481X leads to more accumulation in the nuclei, whereas p.G274C and p.R293H don't affect protein subcellular localization obviously.

### Assessment of Human *GLI1* Wild-Type or Mutant Overexpression on Zebrafish Cardiogenesis

**Figure 5:**
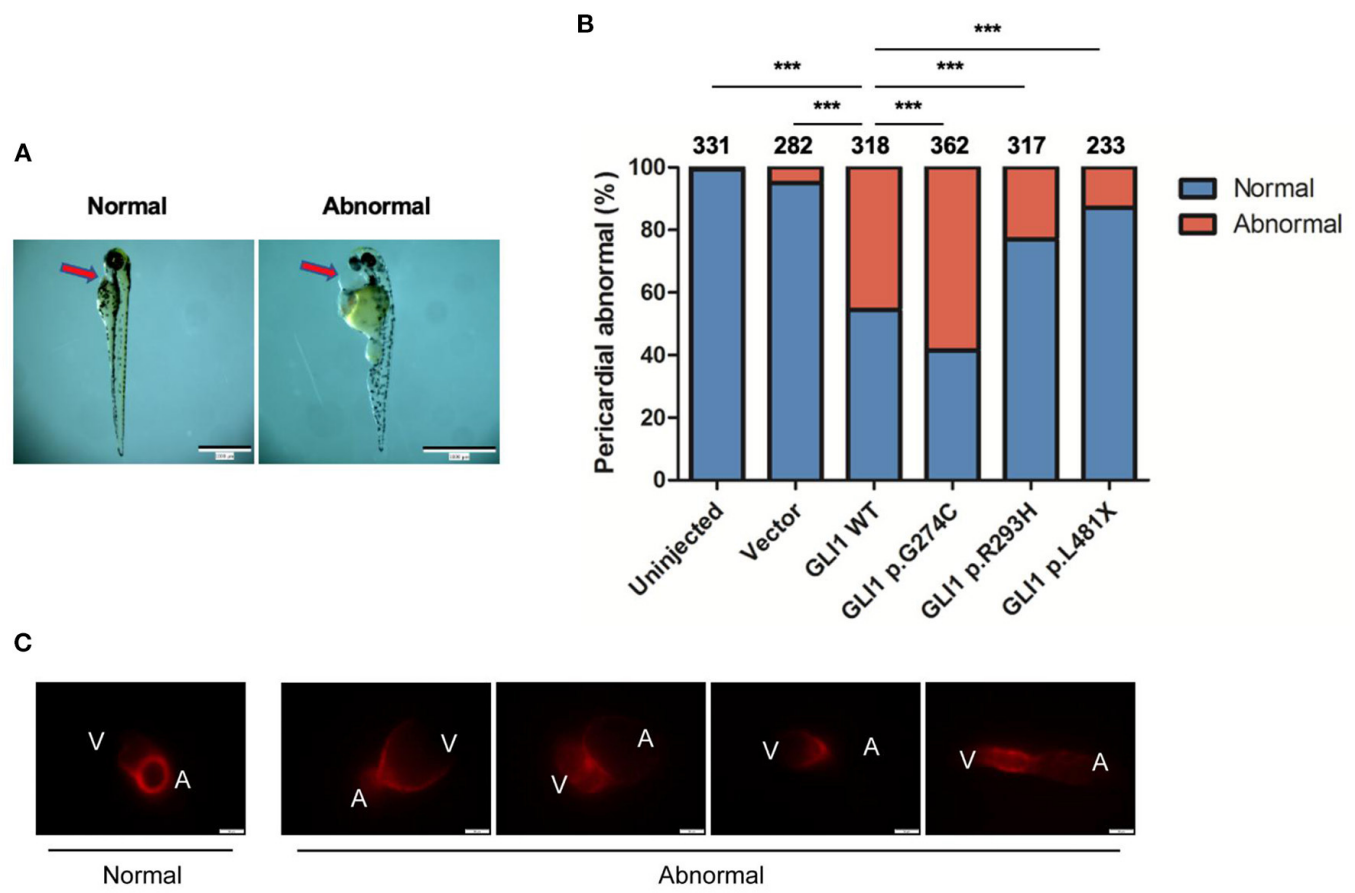
Effect of overexpressed human *GLI1* wild-type or mutant on zebrafish heart development. Empty vector or *GLI1* wild-type/mutant expression construct were injected into 1-2 cell stage fertilized zebrafish embryos, and the teratogenic effect in zebrafish cardiogenesis was calculated 72 h post injection. **(A)** Red arrows indicate the pericardial cavity of zebrafish embryos. **(B)** The frequencies of pericardial abnormalities. (n ≥ 200, *P < 0.05, **P < 0.01, ***P < 0.001, compared with wild-type group). **(C)** Phenotype of Cmlc2-mCherry zebrafish after overexpressing wild-type human *GLI1. GLI1* wild-type expression construct were injected into 1-2 cell stage fertilized Cmlc2-mCherry zebrafish embryos. Images were captured with Olmpus IX83 72 hours post injection. V, ventricle; A, atrium.

The Sonic hedgehog (SHH) signaling pathway is evolutionarily conserved and plays critical roles in organogenesis, particularly of the embryonic heart. Both elevated and repressed core factors in signal transduction will cause failure of heart development as precise spacial and temporal signaling regulation is crucial (38, 39). Hence, we performed plasmid microinjection into one to two cell stage fertilized zebrafish embryos to evaluate the dominant negative teratogenic effect of human *GLI1* overexpression on zebrafish cardiogenesis. Indeed, we observed multiple cardiovascular abnormalities as a result of wild-type human *GLI1* gene overexpression in zebrasish embryos, including malformations on atrium, ventricle, atrioventricular septal and heart blood flow and beat rate (Figure 5C, Supplementary Videos S1–S5). We then calculated the enlarged pericardium (Figure 5A) which is a common phenotype of zebrafish heart malformations (25, 40–42) to evaluate GLI1 function alteration by mutations. As shown in Figure 5B, compared to the uninjected and empty vector groups, p.G274C resulted in the most heart malformation occurence, indicating its enhanced protein function over the wild-type form. Mutations p.R293H and p.L481X generated less abnormal embryos than wild-type GLI1 protein, showed the protein function loss by those two mutations. This result further confirmed that the function of *GLI1* gene was seriously affected by those three mutations.

## Discussion

SHH signaling exists in a variety of animals and plays a fundamental role in regulating accurate organization of the body plan, and its abnormal activation occurs in a variety of tumor cells (17). The GLI proteins are the main effectors of SHH signal and characterized as DNA-binding transcription factors (11, 43). The critical roles of *GLI1-3* genes in embryonic development has been well established and is manifested by the clinical features of patients with mutations in these genes (17, 18). However, the contribution of these genes on human cardiovascular system formation remains less studied. Herein, we conducted genetic screening in CHD patients and identified several case specific nonsynonymous rare mutations.

Previous studies have shown that perturbations of Hedgehog pathway can lead to developmental errors presenting partially overlapping clinical manifestations and atrioventricular canal defects (AVCD) as a common denominator. Moreover, Shh pathway also involved in cardiac outflow tract and neural crest development and, therefore, *Shh*^−/−^ embryos display conotruncal and pharyngeal arch artery defects (44, 45). Some variants of *GLI1-3* genes have been found in several diseases including polydactyly, holoprosencephaly, Pallister-Hall syndrome and others (18, 46–51). However, our CHD patients are sporadic and non-syndromic cases in which ones showing additional symptoms such as polydactyly or holoprosencephaly should be excluded already. All the variants of *GLI1-3* genes we identified are heterozygous, and showed much stronger connection with AVSD and PDA than other subtypes of CHD. While this phenotype-genotype correlation deserves further validation by expanding the sample size in rare mutation study.

Dual-luciferase reporter assay revealed p.G274C mutation enhanced transcription activation, p.R293H decreased transcription activation, whereas p.L481X almost completely loses transcription activation of SHH signaling pathway. EMSA showed p.G274C increased DNA-binding affinity and so did the mutant p.R293H. However, the luciferase assay showed that mutant p.G274C gains stronger function and mutant p.R293H decreases activation of the signaling compared to the function of wild GLI1 protein. Then we defined mutant p.G274C as gain-of-function and mutant p.R293H as loss-of-function. The fact that p.R293H shows loss-of-function does not necessarily need to be associated with decreased DNA-binding ability. The mutation specific characteristics may determine that its impact on the reduction of function is much greater than its impact on the ability to bind DNA. Therefore, the comprehensive performance of mutant p.G293H is that the function is reduced and the DNA-binding ability is reversely increased. Alternatively, the loss-of-function gained by mutant p.G293H might be too prominent to be balanced by the reverse effect of increased DNA-binding ability.

Usually, stop-gain mutations with aberrant translation termination leads to the degradation of the mRNA via activation of nonsense-mediated mRNA decay (NMD) and fail to produce a stable truncated protein (52). In response to NMD of mutated allele, wild-type allele always increasingly expressed to avoid haploinsufficiency. However, our data have demonstrated that the L481X truncated protein can exist stably and the truncated protein is largely produced *in vivo* (Supplementary Figure S3). The truncated protein not only failed to activate SHH signaling, but also placed additional inhibitory effects on SHH target genes (Figures 2, 4). Therefore, mutation p.L481X resulting haploinsufficiency was supported by the evidence that the heterozygous mutation p.L481X caused reverse phenotypes when the wild-type allele expression was strictly limited in half by the existed truncated allele. Because the result that heterozygous mutation p.L481X caused reverse phenotypes was partially attributed to the truncated allele, it's also hard to conclude that what we observed was merely caused by the half-expressed wild-type allele.

The alterations of critical genes in the early stage of cardiac development, such as *de novo* mutations, copy number variants, common variants, noncoding mutations and epigenetic modification, have contributed to the occurrence of CHD (5, 53–55). The *in vitro* function assays indicated that compared to the normal function of wild-type GLI1 protein, mutation p.R293H shows loss of function effect and mutation p.L481X also causes severely loss-of-function effect due to the stop-gain, while, mutation p.R274C shows gain of function. When we used overexpression system to test the cardiac morphology in zebrafish, as expected, the overexpressed wild-type *GLI1* gene would lead to observed cardiac anomalies because of the dosage imbalance in addition to the existed endogenous *GLI1* working normally. Similarly, compared to the wild-type GLI1 protein, the overexpressed p.R293H mutated GLI1 or p.L481X mutated GLI1 protein would result in less reverse effect *in vivo* because they are partially or maximum lose the normal function as wild-type GLI1 protein. Supposed mutation p.R274C gains of the original function, the most severe reverse effect on zebrafish phenotypes would be imaginable. In other words, the *in vitro* and *in vivo* function assays kept consistent although the outcomes looked a sign of contradiction.

Our functional study demonstrated some of those mutations significantly interfere with the protein function, thus lead to dysregulated SHH signaling pathway. Our study suggests the strong association of *GLI1* gene mutations with the CHD occurrence. Interestingly, both gain and loss-of-function nonsynonymous rare mutations in *GLI1* gene were identified in the CHD patients. As GLI1 protein simply serves as a transcriptional activator in SHH signaling pathway, our results suggest that either elevated or repressed GLI1 protein activity caused imbalanced signaling transduction could be a causative factor of CHD. This fits the fact that temporally and spatially tight regulation of key factors in fundamental developmental pathways underlies the normal embryogenesis.

However, since our samples are all sporadic and lack pedigrees, the mutations we found might not be the only causative factor leading to CHD. It is possible that the patients with *GLI1-3* mutations may also carry harmful mutation(s) in other gene(s). We presume that single deleterous mutation contributes partially to the CHD and multiple such mutations help individual approaches the threshold of CHD occurrence, as our group demonstrated in another congenital malformation recently (56).

In conclusion, in present study, we systematically identified a set of novel rare mutations in *GLI1-3* genes in human CHD that have the potential to be used diagnostically. Our data support our hypothesis that in *GLI1*, both gain and loss-of-function mutations are likely to be associated with CHD. Further understanding of the regulation and balance of SHH signaling pathway is crucial for understanding CHD etiology.

## Data Availability Statement

The data in the present study are available from the corresponding authors upon reasonable request. The raw data supporting the conclusions of this article will be made available by the authors, without undue reservation.

## Ethics Statement

The studies involving human participants were reviewed and approved by Ethics Committee of the School of Life Sciences, Fudan University. Written informed consent to participate in this study was provided by the participants' legal guardian/next of kin. The animal study was reviewed and approved by Ethics Committee of the School of Life Sciences, Fudan University.

## Author Contributions

RP, BL, LL, and HW designed the project and prepared and edited the manuscript. RP, BL, and LL performed the experiments and analyzed the data. SC, YG, and XY contributed to the zebrafish embryo injection experiment. ZS and LY contributed to the clinical specimens collection and sequencing. All authors contributed to the article and approved the submitted version.

## Funding

This work was supported by grants from National Key R&D Program of China (2021YFC2701101, HW) and National Natural Science Foundation of China (81930036 and 82150008, HW) and the Commission for Science and Technology of Shanghai Municipality (20ZR1404800, LL).

## Conflict of Interest

The authors declare that the research was conducted in the absence of any commercial or financial relationships that could be construed as a potential conflict of interest.

## Publisher's Note

All claims expressed in this article are solely those of the authors and do not necessarily represent those of their affiliated organizations, or those of the publisher, the editors and the reviewers. Any product that may be evaluated in this article, or claim that may be made by its manufacturer, is not guaranteed or endorsed by the publisher.
