# Supplementary material for: Deleterious Rare Mutations of GLI1 Dysregulate Sonic Hedgehog Signaling in Human Congenital Heart Disease
Source: Front Cardiovasc Med. 2022 Apr 4;9:798033. doi: 10.3389/fcvm.2022.798033 (PMC9014293; doi:10.3389/fcvm.2022.798033)
Supplement: Supplementary file 1 [file Data_Sheet_1.pdf]

SUPPLEMENTARY MATERIAL

TABLE S1 | Primers of Sanger DNA sequencing for confirmation the variants.

TABLE S2 | PCR primers of GLI1-3 ORF.

TABLE S3 | Probes for electrophoretic gel mobility shift assay (EMSA).

FIGURE S1 | The sequencing chromatogram of Sanger sequencing for confirmation the variants.

FIGURE S2 | Conservative analysis of GLI1-3 mutations in CHD patients. Partial alignment of GLI1-3 amino acid among Homo Sapiens (P08151), Pan Troglodytes (A0A2I3SME4), Sus scrofa (F1SKG0), Rattus Norvegicus (G3V6X8) and Mus Musulus (P47806) generated by UniProt online alignment.

FIGURE S3 | Protein stability was not affected by GLI1 mutations. HEK 293T cells were transfected with HA-tagged wild-type or mutant GLI1 expression constructs (GLI1 p.L481X was truncated including aa 1-480 with C-terminal HA-tag). Whole cell lysate were used in Western blot assay using anti-HA antibody. GAPDH serves as internal control.

VIDEO S1-5 | Zebrafish heart blood flow and beat rate after overexpressing wild-type human *GLI1*. S1: Normal; S2-5: Abnormal.

TABLE S1 | Primers of Sanger DNA sequencing for confirmation the variants.

primer-ID	primer sequence (5' to 3')
GLI1 p.G274C F	AGTCGTGGAAGAGGAACAGG
GLI1 p.G274C R	CGTGCCTGGCCTGTTTTAAT
GLI1 p.R293H F	AGTCGTGGAAGAGGAACAGG
GLI1 p.R293H R	CGTGCCTGGCCTGTTTTAAT
GLI1 p.L481X F	CAAGTTGAAGGAGCTGTGGG
GLI1 p.L481X R	AGGGTGACTTCCTCCTCTCA
GLI1 p.R592S F	TCTGCCTATACTGTCAGCCG
GLI1 p.R592S R	TCTAGCATCCATGGCAGCAT
GLI1 p.M617I F	TCTGCCTATACTGTCAGCCG
GLI1 p.M617I R	TCTAGCATCCATGGCAGCAT
GLI1 p.P642S F	TCTGCCTATACTGTCAGCCG
GLI1 p.P642S R	TCTAGCATCCATGGCAGCAT
GLI2 p.H78P F	TTTGTCAAGAGCGCAGTTGG
GLI2 p.H78P R	TCCCAGGCCATGATAGCAAA
GLI2 p.A391V F	ATGGTCTGTGCGGAGAGATC
GLI2 p.A391V R	AGCCTGAGACAAGAGGGATG
GLI2 p.V681M F	TACCCTCTGAGTCTGAGCCT
GLI2 p.V681M R	TCTCACCCCTGCCATTGATT
GLI2 p.G716V F	TACCCTCTGAGTCTGAGCCT
GLI2 p.G716V R	TCTCACCCCTGCCATTGATT
GLI2 p.K736N F	TACCCTCTGAGTCTGAGCCT
GLI2 p.K736N R	TCTCACCCCTGCCATTGATT
GLI2 p.N755S F	TACCCTCTGAGTCTGAGCCT
GLI2 p.N755S R	TCTCACCCCTGCCATTGATT
GLI2 p.A852T F	GTGTTGCAAGCCCTCTTCTC
GLI2 p.A852T R	GTTGTGGGTGCTGTGGAAG
GLI2 p.A1113V F	CTTCCACAGCACCCACAAC
GLI2 p.A1113V R	CACCTCATTCCACTGCACAG
GLI2 p.P1290L F	GTCCTGGCATGACTACCACT
GLI2 p.P1290L R	CCTCTGCTGTTTCCTGACTG
GLI2 p.I1451S F	CTCACCCAGTCCAGAGCTAC
GLI2 p.I1451S R	GCATCGAAGTCAATCTGGGG
GLI3 p.P75A F	GCCAACCCCTACATCTCAGT
GLI3 p.P75A R	CCTCCCCATAGCTCCTGAAC
GLI3 p.P169L F	GCCTCTGCCTGTGGAGATAT
GLI3 p.P169L R	GCTGCATGATCTCCAGAAGC
GLI3 p.A286V F	TCTAAGCAGCCGTCAGATTCA
GLI3 p.A286V R	CCTCTTGGTATAGGCACAGC
GLI3 p.V1097L F	TGCTTCAGAATTACACGCGG
GLI3 p.V1097L R	ACTTGAGCTTGGAGGAGGAC

TABLE S2 | PCR primers of GLI1-3 ORF.

primer-ID	primer sequence (5’ to 3’)
GLI1-F(SgfI)	GAGGCGATCGCATGTTCAACTCGATGACCCC
GLI1-R(MluI)	CGACGCGTGGCACTAGAGTTGAGGAATT
L481X F(SgfI)	GAGGCGATCGCATGTTCAACTCGATGACCCC
L481X R(Mlu)	CGACGCGTGCTGGAGAGGTCTTCAGTGC
GLI2-F(SgfI)	GAGGCGATCGCAATGGAGACGTCTGCCTCAGC
GLI2-R(NotI)	ATAAGAATGCGGCCGCGTGGTCATCATGTTCAGGAACT
GLI3-F(SgfI)	GAGGCGATCGCAATGGAGGCCCAGTCCCACAG
GLI3-R(MluI)	CGACGCGTTTGCATAACTGCAAGGAATT

TABLE S3 | Probes for electrophoretic gel mobility shift assay (EMSA).

probe-ID	probe sequence (5' to 3')
GLI consensus probe F	Biotin-AGCTACCTGGGTGGTCTCT
GLI consensus probe R	Biotin-TCGAAGAGACCACCCAGGT
GLI consensus F	AGCTACCTGGGTGGTCTCT
GLI consensus R	TCGAAGAGACCACCCAGGT
Non-competitive oligo F	AGGAGCTCGTGCAGACCAA
Non-competitive oligo R	TTGGTCTGCACGAGCTCCT

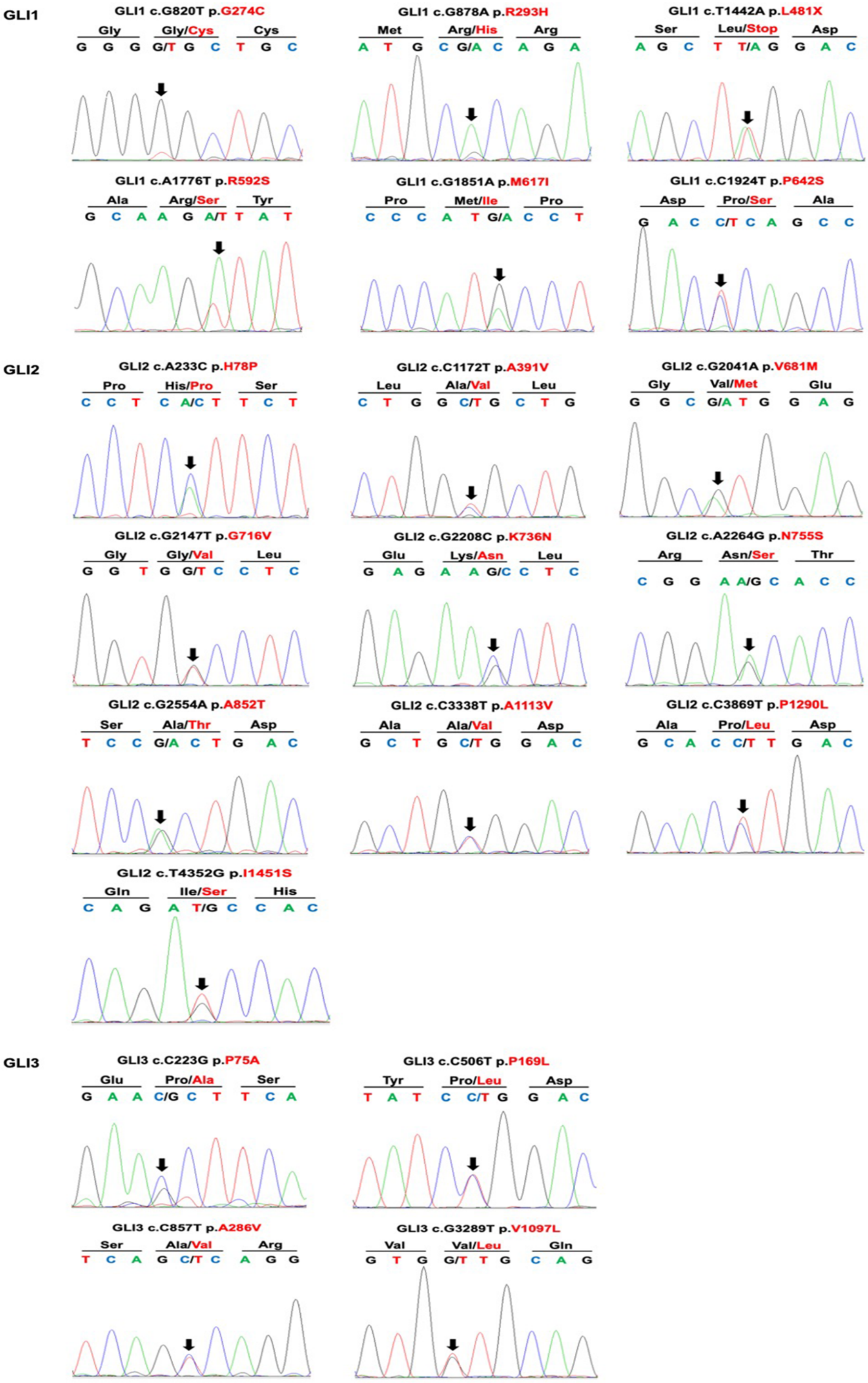

FIGURE S1 | The sequencing chromatogram of Sanger sequencing for confirmation the variants.

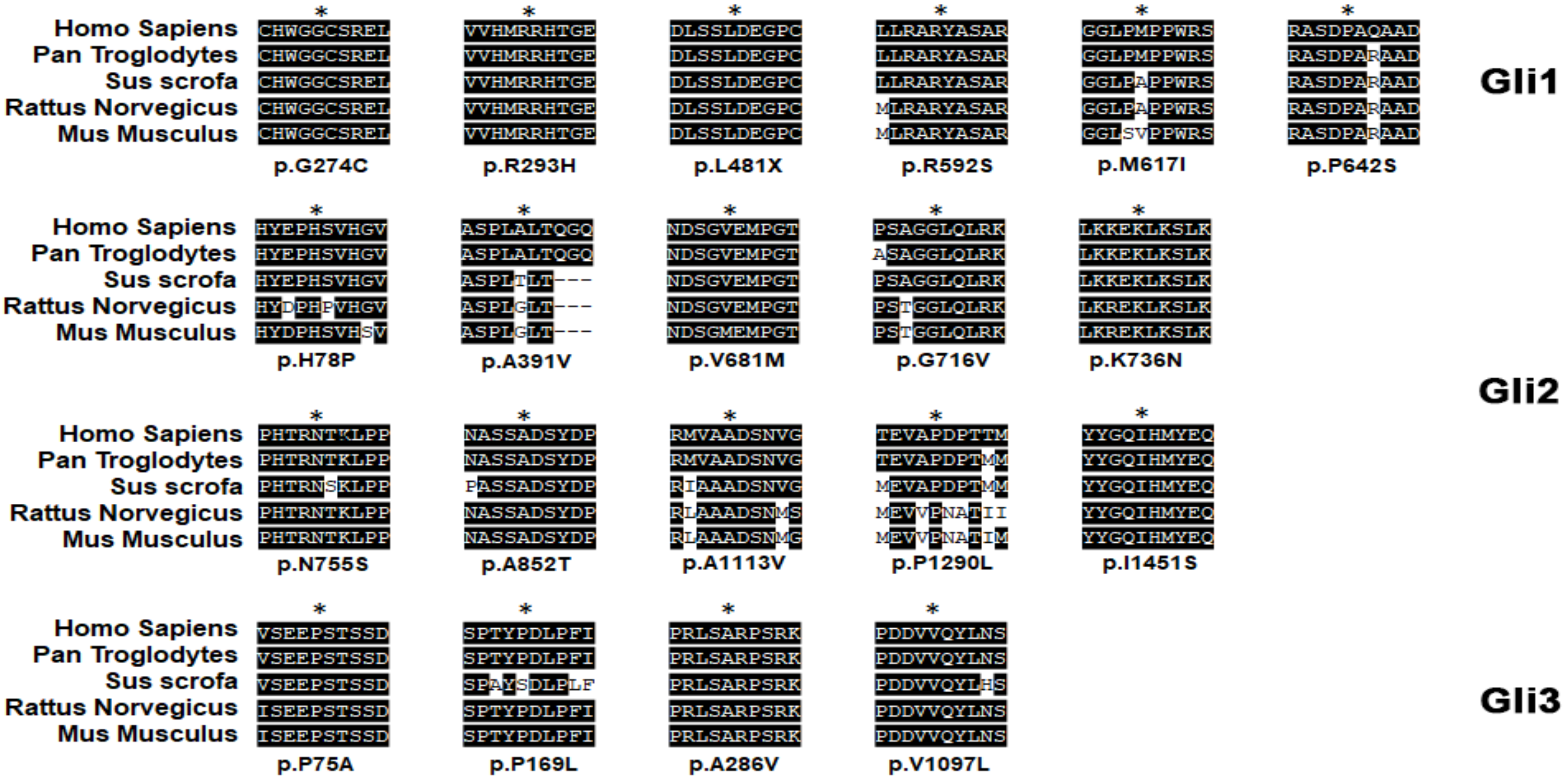

FIGURE S2 | Conservative analysis of GLI1-3 mutations in CHD patients.

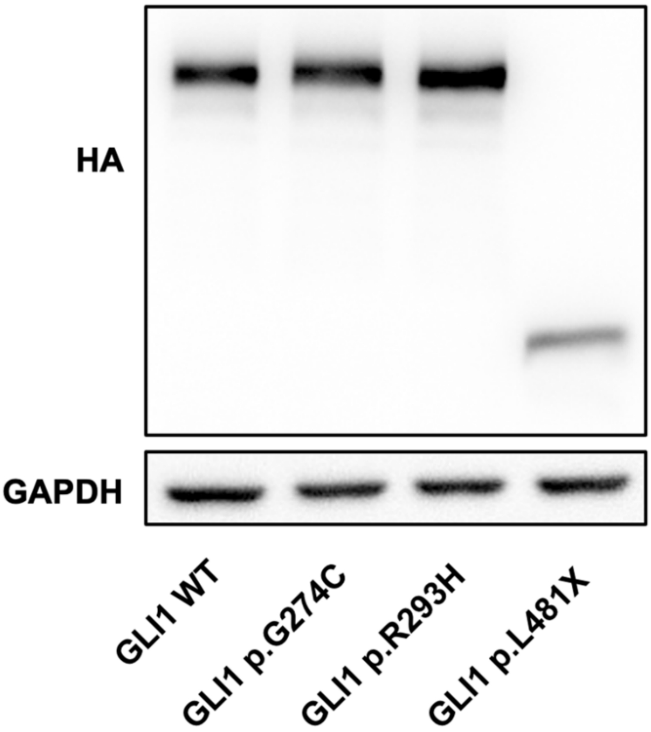

FIGURE S3 | Protein stability was not affected by GLI1 mutations.
